# The Role of Matrix Metalloproteinases in Endometriosis: A Potential Target

**DOI:** 10.3390/biom11111739

**Published:** 2021-11-22

**Authors:** Junya Ke, Jiangfeng Ye, Mingqing Li, Zhiling Zhu

**Affiliations:** 1 Department of Obstetrics and Gynecology, Obstetrics and Gynecology Hospital of Fudan University, Shanghai 200011, China; 17211250006@fudan.edu.cn; 2 Department of Integrated Traditional & Western Medicine, Obstetrics and Gynecology Hospital of Fudan University, Shanghai 200011, China; 3 Institute of Obstetrics and Gynecology, Obstetrics and Gynecology Hospital of Fudan University, Shanghai 200011, China; 4 Division of Obstetrics and Gynecology, KK Women’s and Children’s Hospital, Singapore 229899, Singapore; ye.jiangfeng@kkh.com.sg; 5 Shanghai Key Laboratory of Female Reproductive Endocrine-Related Diseases, Shanghai 200011, China

**Keywords:** matrix metalloproteinases, endometriosis, function, potential value

## Abstract

Endometriosis is a condition that is influenced by hormones and involves stroma and glands being found outside the uterus; there are increases in proliferation, invasion, internal bleeding, and fibrosis. Matrix metalloproteinases (MMPs) have been suggested to be crucial in the progression of invasion. The MMP family includes calcium-dependent zinc-containing endopeptidases, some of which not only affect the process of cell invasion but also participate in other physiological and pathological processes, such as angiogenesis and fibrosis. MMPs act as downstream-targeted molecules and their expression can be regulated by numerous factors such as estrogen, oxidative stress, cytokines, and environmental contaminants. Given their unique roles in endometriosis, MMPs may become effective biomarkers of endometriosis in the future. In the present review, we summarize the current literature on MMPs regarding their classification, function, and potential value for endometriosis, which may contribute to our knowledge of MMPs and MMP-targeted interventions.

## 1. Introduction

Endometriosis is a benign, hormone-dependent disease characterized by the presence of endometrial glands and stroma outside the uterus and affects at least 10–15% of women of reproductive age [1]. Several characteristics, such as infertility, dysmenorrhea, and dyspareunia, are associated with endometriosis [2]. Moreover, estrogen, matrix remodeling, inflammation, and oxidative stress have been shown to be involved in endometriosis progression from early to advanced stages [3].

Based on the hypothesis of the retrograde transplantation theory proposed by Sampson in 1927, we recognize the significance of matrix regrading and matrix remolding. Endometriosis is established when endometrial cells (influenced by hormone fluctuations) break off and travel via the fallopian tubes to new sites, where they implant and grow. The extracellular matrix (ECM) is a complex network of macromolecular structures, such as collagens, proteoglycans, glycoproteins, and elastin [4]. The matrix metalloprotease (MMP) family is devoted to maintaining ECM homeostasis, and the dysregulation of its expression leads to the disease. Since the 1960s, the MMP family has drawn much attention, and its functions have been further determined [5] and systematically reviewed. [6].

In this review, we propose a role for MMPs in endometriosis and describe their different functions in various biological processes, such as invasion, angiogenesis, and fibrosis. More importantly, we further discuss the potential treatment options for endometriosis, especially anti-MMP interventions.

## 2. The MMP Family

MMPs belong to a large family of calcium-dependent, zinc-containing endopeptidases that are known for their ability to cleave nonmatrix proteins, as well as several ECM constituents (e.g., collagens, proteoglycan, and glycoproteins). In 1949, MMPs were discovered to be depolymerizing enzymes that could promote the proliferation of malignant cells by remolding connective tissue stroma such as blood vessels [7]. A decade later, the first vertebrate MMP was isolated and identified to act as a collagenase [5]. At least 28 MMPs have been identified in mammals to date [8].

### 2.1. Classification of MMPs

How MMPs should be classified remains controversial. A previous report classified them into the following five groups based on their substrate-specificity and location: collagenases, gelatinases, stromelysins, matrilysins, and membrane-type metalloproteinases [9]. However, Garcia-Fernandez et al. and Kapoor et al. both divided MMPs into six groups depending on their different substrates. Compared with the previous five groups, the latter researchers added an “other MMPs” group, which included those that did not fit into any other category [7,10]. Beyond that, MMPs can also be divided into two groups: secreted MMPs and anchor MMPs. Of the known MMPs, MMP14, 15, 16, 17, 23, 24, and 25 are membrane-anchored [11,12].

### 2.2. Structure of MMPs

The sophisticated structure of MMPs has been known for some time and has been described in significant detail [13]. The compositions of most MMPs are the same: they involve a pro-peptide domain, a signal peptide, a cysteine switch motif, a catalytic domain, and a hemopexin-like domain [7]. However, some MMPs have different structural characteristics that enable them to perform special activities. For example, MMP2 and MMP9 can interact with collagen via three fibronectin type-II domains. Moreover, MMP7, MMP23, and MMP26 have no C-terminal hemopexin-like domain, which usually consists of 190 amino acids. Additionally, MMP23 is the only MMP that possesses a cysteine-rich and Ig domain. Some researchers have designed MMP inhibitors according to the specific structures. Ilomastat (GM-6001), a first-generation collagen peptidomimetic, is a broad-spectrum MMP inhibitor [14,15]. However, due to its poor bioavailability, clinical trials of ilomastat have failed. However, Tanomastat (BAY 12-9566) has been shown to potently and selectively inhibit MMP13, gelatinase A, and gelatinase B. It is an analog of biphenyl non-peptide butanoic acid and was first developed by Bayer, Inc. (Leverkusen, Germany) [16]. However, no anti-MMP inhibitors with few side effects and strong specificity have been used in clinical anti-tumor therapy to date.

### 2.3. The Expression of MMPs (Natural MMP Inhibitors)

MMPs are generally poorly expressed in humans due to their specific endogenous inhibitors, known as the tissue inhibitor of metalloproteinase (TIMP) family, which includes four known proteins: TIMP-1, 2, 3, and 4 [17]. TIMPs can bind to the catalytic domains of MMPs with a 1:1 stoichiometric ratio and then block their enzymatic activity [18,19]. The TIMP family includes proteins with specific substrates; for example, TIMP-1 can only regulate the membrane-type MMPs, but the other three members have wider ranges of biological activity [9]. In addition to MMPs, TIMPs can suppress other enzymes, such as those of the disintegrin and metalloproteinase (ADAM) family [20,21,22].

## 3. MMP Expression in Endometriosis

### 3.1. Summary of Clinical Studies

Aside from MMP7, which is only expressed in epithelial endometrial cells, MMPs are present in both the stromal and epithelial tissue compartments of the endometrium [23]. As an invasive disorder, endometriosis involves increased MMP activity. Several studies have reported that the levels of MMPs are elevated in the ectopic tissue, peritoneal fluid, or sera of patients with endometriosis, especially MMP2 and MMP9 [24,25,26]. In addition, Borghese et al. shared gene expression profiles of eutopic. vs. ectopic endometrium in 2008 and provided a list of more than 5600 genes related to endometriosis. The overexpressed extracellular matrix genes (such as MMP23 and MMP26) showed significant expression differences [27]. However, the potential mechanism of the dysregulation of MMP23 and MMP26 expression still needs further research. Considering their key roles in endometriosis, MMPs have been considered potential therapeutic targets for this disease. As presented in Table 1, accumulating evidence has demonstrated that MMPs play significant roles in promoting the development of endometriosis.

### 3.2. Complexity of MMP Regulation in Endometriosis

Over the past few years, evidence has shown that MMPs play an important role in the mechanisms involved in the occurrence and treatment of endometriosis. The epidermal growth factor receptor (EGFR)-MMP7 signaling pathway has been shown to be involved in the regulation of epithelial-mesenchymal transition (EMT) during the progression of endometriosis [45]. Moreover, chloride channel-3 (CIC3) and stress-induced phosphoprotein 1 enhance the activity of MMP9, while microRNA-33b has the opposite effect [48,49,50]. The fibrinogen alpha chain is upregulated and affects MMP2 in endometriosis [51]. Moreover, it has been shown that leptin promotes cell migration and invasion and that the cyclooxygenase-prostaglandin E2 (PGE2)-pAKT axis can promote angiogenesis via MMP2 [37,52]. In an in vivo trial, Shu et al. observed that the silencing of aquaporin 1, a water-channel protein, could influence the expression of invasion-related factors (MMP2, MMP9, TIMP1, and TIMP2), alleviating the progression of endometriosis in a mouse model [53]. Lipoxin A4 (LXA4) is a lipid medium that is widely involved in the establishment of endometriosis [54,55]. Moreover, LAX4 can suppress estrogen-mediated EMT via binding to its receptor and can inhibit the activities of MMP2 and MMP9 [56].

**Figure 1:**
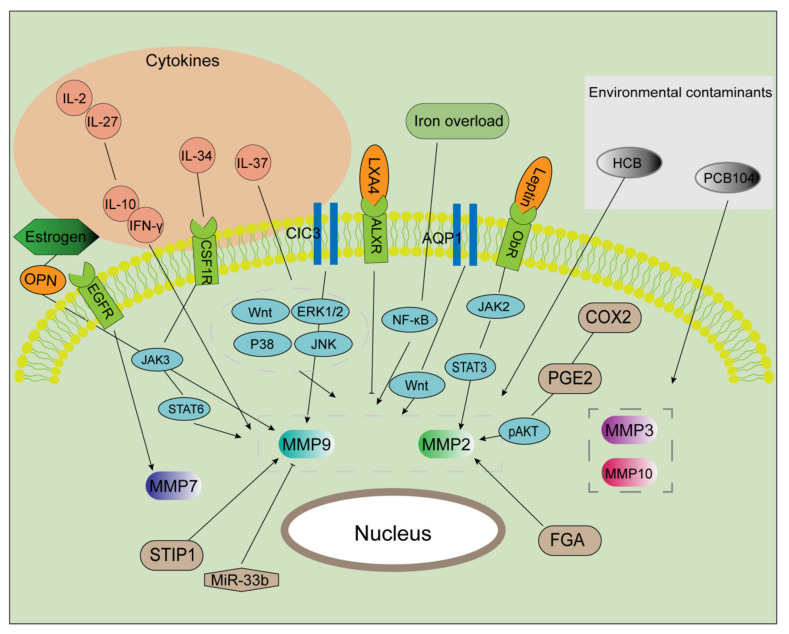
Multiple factors regulate MMP activities. After exposure to certain environmental contaminants (e.g., PCB104 and HCB), the expression of MMPs (MMP3, 10, 2, and 9) is markedly enhanced. IL-37 upregulates the expression of MMPs via multiple signaling pathways. IL-2 and IL-27 were found to maintain the balance of IL-10 and IFN-γ, promoting MMP2 and MMP9 expression and then inducing cell invasion and proliferation. IL-34 binds to CSF1R, which activated the JAK/STAT6 pathway in an autocrine manner. Estrogen induces MMP9 expression via the OPN. CIC3 and STIR1 improve the activity of MMP9, while miR-33b inhibits it. AQP1 promotes the expression of MMP2 and 9 via the Wnt signaling pathway. The COX2/PGE2/pAKT axis, as well as the leptin/JAK2/STAT3 axis, serves as a significant regulator in increasing MMP2 expression. Additionally, MMP2 is a target of FGA and LXA4. MMP7 is a downstream component in the EGFR-mediated signaling pathway. Iron markedly increases EMT and MMP2/9 activities in endometriosis.

It is well known that the microenvironment of patients with endometriosis is inflammatory. Interleukin (IL)-2 and IL-27 synergistically inhibit MMP9 expression by maintaining the balance of interferon (IFN)-γ and IL-10, thereby improving the invasive ability of endometriosis cells [57]. Lin et al. reported that IL-34, through activating signal transducer and activator of transcription 6 (STAT6), promoted the expression of MMP9 in endometriosis in vitro and in vivo via the colony-stimulating factor 1 receptor/Janus kinase 3/STAT6 pathway [58]. Moreover, IL-37 affects downstream MMP9 expression via a variety of signaling pathways and regulates the biological behavior of endometrial stromal cells [59]. MMP2 and MMP9 can be regarded as the most typical downstream biomarkers in the progression of endometriosis. Furthermore, as shown in Figure 1, various extracellular factors, such as estrogen [60], cytokines [57,58,59], iron overload [61], and environmental contaminants [62,63], contribute to the regulation of MMPs expression.

MMP, matrix metalloproteinase; PCB104, polychlorinated biphenyl 104; HCB, hexachlorobenzene; CSF1R, colony-stimulating factor 1 receptor; OPN, osteopontin; CIC3, chloride channel-3; STIR1, stress-induced phosphoprotein 1; miR, microRNA; AQP1, aquaporin 1; FGA, fibrinogen alpha chain; COX2, cyclooxygenase 2; PGE2, prostaglandin E2; p, phosphorylated; JAK2, Janus kinase 2; STAT3, signal transducer and activator of transcription 3; LXA4, lipoxin A4; EMT, epithelial-mesenchymal transition.

## 4. The Role of MMPs in the Pathophysiology of Endometriosis

**Figure 2:**
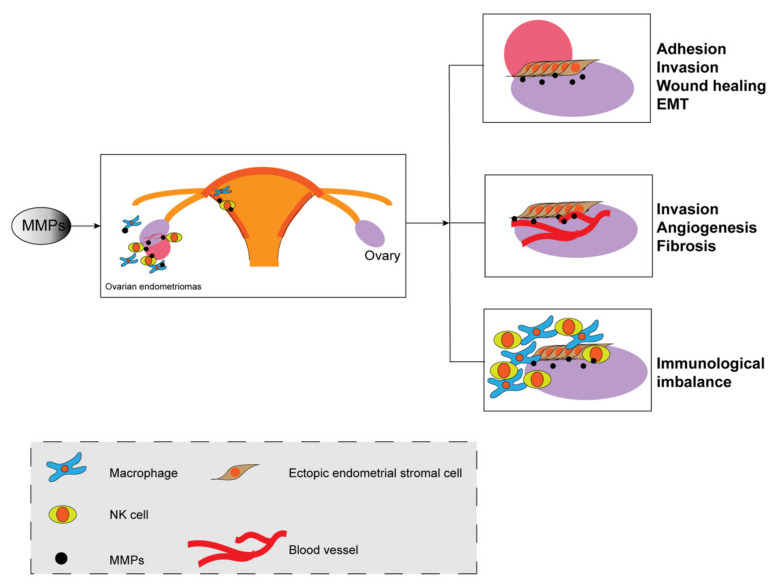
The role of MMPs in the pathophysiology of endometriosis.

MMPs are influenced by changes in steroid hormone concentration levels and are involved in cyclic changes of the endometrium’s structure and thickness [6]. The endometrium, whether it is eutopic endometrium or ectopic endometrium, is periodically shed in response to hormone fluctuations, but in endometriosis, it changes at the cellular level, e.g., in epithelial-mesenchymal transition (EMT), cell migration, and invasion, and at the tissue/organism level, e.g., in angiogenesis, fibrosis, and immunological aspects (Figure 2). There is no doubt that MMPs play a vital role in this process.

In endometriosis, MMPs play a key role in various pathophysiological processes. The endometrium is shed during menstruation due to the fluctuation of hormones, and MMPs then accelerate the growth of the new endometrium to cover the wound. Free endometriotic cells pass through the fallopian tube; adhere to the surfaces of the peritoneum, ovary, and other organs; degrade the extracellular matrix; invade the new site; promote angiogenesis. Immunocytes, such as macrophages, kill the free cells upon coming into contact with them. MMPs are crucial for the aforementioned processes.

### 4.1. MMPs at the Cellular Level

#### 4.1.1. Migration and Invasion

Endometriosis is a common benign gynecological disease characterized by a high migratory and invasive potential. In order to migrate, invade, and grow in new places, the cells establish cell-cell or cell-ECM interactions. In a study of the endometrial stromal cell line St-T1b and primary endometriotic stromal cells, researchers found that they participated in directional migration with significant collagen I remodeling, and this behavior was inhibited by the broad-spectrum MMP inhibitor N-isobutyl-N-(4-methoxyphenylsulfonyl) glycyl ydroxamic acid (NNGH) [64]. This confirms that MMPs are involved in ECM remodeling which is required for the establishment of ectopic endometriosis lesions. This initial step of lesion formation requires MMP activity for basement membrane and ECM-protein breakdown to support the subsequent invasion of endometriosis cells into the peritoneum [6,65]. The first membrane-type MMP to be named was MT1-MMP (also referred to as MMP14), which can directly degrade the ECM, especially collagen I, by locating invadopodia [66]. As downstream target molecules in the complex network that regulates endometriosis invasion, MMPs are altered by a diverse range of substances. For instance, the cytokines IL-2 and IL-27 promote the expression of MMPs, enhancing invasion by maintaining the homeostasis of IL-10 and IFN-γ [57]. Moreover, exposure to bisphenol A (BPA) exposure increases cell invasion (through MMP2 and MMP9) in a dose-dependent manner, as shown by an in vitro study [67]. In addition to cytokines [57,58] and environmental contaminants [67,68], estrogen [56,69,70,71], oxidative stress [71,72], and autophagy [73,74] all impact invasion. An increasing number of researchers have explored inhibiting cell invasion or the metastasis of endometriosis by decreasing MMP expression. These findings have shown that MMPs are involved in the occurrence and progression of endometriosis by enhancing the invasion of ectopic endometrial cells.

#### 4.1.2. Epithelial-Mesenchymal Transition

When cells lose their epithelial characteristics and acquire the features of mesenchymal cells, this is known as “epithelial-mesenchymal transition”. This process includes a loss of polarity, impaired cell adhesion, and the acquisition of the ability to migrate. Several MMPs have been found to be involved in various cancers, such as gastric cancer, colorectal cancer, ovarian cancer, and prostate cancer [75,76,77,78]. When the expression of Par3 (a marker of cell polarity) and occludin (a tight-junction protein) decreases, the polarity of epithelial cells and the tight junctions between cells decrease [79,80], and then, there is an increase in the migration and invasion abilities [81,82]. These features are beneficial for the formation and development of the new lesion through epithelial-mesenchymal transition. Chatterjee et al. reported that MMP7 promoted epithelial-mesenchymal transition in ovarian endometriosis, and EGF upregulated the expression of MMP7 through the ERK1-AP1 pathway [45]. MMP14 regulates the function and formation of invadopodia, which controls the migration and invasion abilities of mesenchymal cells [83]. Therefore, we speculate that MMPs may play a key role in regulating the EMT process in endometriosis. However, since there are few in-depth studies on the relationship between MMPs and epithelial-mesenchymal transition in endometriosis, this needs to be further explored.

### 4.2. MMPs at the Tissue/Organism Level

#### 4.2.1. Angiogenesis

Angiogenesis is activated after the invasion. Moreover, the establishment and development of ectopic lesions require vasculogenesis for the lesions to be maintained. Angiogenesis is an invasive process initiated by MMPs [84] that contributes to endothelial cells detaching and migrating into new sites. In humans, at least 14 MMPs have been found in the vascular endothelium [12]. Specifically, MMP1, 8, and 13, which belong to the collagenases, are associated with angiogenesis. MMP1 promotes the expression of vascular endothelial growth factor (VEGF) receptor 2 (VEGFR2) [85], while MMP7 promotes angiogenesis by activating the VEGF pathway and degrading VEGFR1 [86]. Similarly, TIMP can regulate angiogenesis by inhibiting neovascularization [87]. Interestingly, MMPs are not only associated with angiogenesis but can also, in turn, be activated by angiogenic factors, such as VEGF and fibroblast growth factors (FGFs) [8].

One analogy is that the “ECM” is a reservoir filled with diverse factors, including VEGF, transforming growth factors, FGFs, and proteases [88,89]. The “ECM” here, unlike the aforementioned ECM that is rich in collagens and glycoproteins, but the so-called “non-ECM” contains growth factors and cell adhesion molecules. This explains why MMPs participate in angiogenesis. MMPs can release various factors by degrading non-ECM. This forms a vicious cycle that promotes the progression of endometriosis, and MMPs may be an indispensable factor among these processes.

#### 4.2.2. Fibrosis

Vigano et al. challenged the obsolete definition of endometriosis and placed fibrosis or myofibroblasts in the public eye [90]. These authors suggested that altering the new definition could greatly reduce the misjudgment of endometriosis and redirect current treatments in a more effective direction. In terms of the real role of MMPs in fibrosis, a previous study revealed the effect of BPA on endometriosis, especially in collagen accumulation. The results demonstrated that exposure to BPA contributed to fibrosis by increasing the synthesis of collagen I and III and decreasing MMP2 and MMP14 expression [91]. Beyond that, another study reported that MT1-MMP deficiency caused the fibrosis of soft tissue because of a reduction in collagen degradation [92]. Interestingly, the findings of Matsuzaki et al. were the opposite. These authors observed that increased matrix stiffness promoted not only collagen I synthesis, but also MMP1 and MMP14 expression [93]. These results can be attributed to the fact that there may be a precise balance between collagen synthesis and degradation that should be explored in the future. Despite this, it is undeniable that MMPs play a vital role in the formation of collagen, which is important for the gradual fibrosis of endometriosis.

#### 4.2.3. Immunological Imbalance

Endometriosis is a chronic inflammatory disorder. Several studies have analyzed numerous cytokines, chemokines, and immunocytes that are altered in endometriosis [94,95,96]. Oosterlynck et al. found that the activity of natural killer (NK) cells was decreased in females with endometriosis [97]. Notably, microenvironment-associated ECM components regulate NK cells’ functions. MMPs can help tumor cells evade immune surveillance via the cleavage of NK group 2 member D (NKG2D) ligands from cell surfaces [98,99]. Additionally, they can cause the shedding of intercellular-adhesion molecule 1, a protein that participates in the process of NK cells’ recognition of their target cells [95,100]. Macrophages, acting as another scavenger in endometriosis, are also impaired. The reduced expression of MMPs on macrophages may be associated with their impaired phagocytic activity, as MMPs can help the macrophages to degrade the ECM of target cells that will be phagocyted [101]. Wu et al. suggested that the levels and activities of MMP9 in peritoneal macrophages were decreased and regulated by prostaglandin (PG) E2 via the EP2/EP4 pathway. Collectively, this may be a potent mechanism of the decrease in the phagocytotic capability of macrophages [102]. The cytokines detected in the peritoneal fluid show different expressions and may be involved in regulating MMPs. IL-10 and IFN have the ability to increase the expression of MMP2 and MMP9 [57], while IL-6 can upregulate the expression of MMP9 [103], and IL-β can positively adjust MMP13 activity [104]. Overall, it has been suggested that MMPs may cause an imbalance in local microenvironmental immunity in endometriosis and induce the occurrence of disease, by modulating immune cells or autoimmune factors.

As previously mentioned, endometriosis is a complex disease that is regulated by numerous interacting factors which interact. Similarly, the involvement of MMPs is not limited to the aforementioned stages in the progression of endometriosis, and their additional functions require further investigation.

## 5. Potential Value of Inhibiting MMPs in Endometriosis

### 5.1. MMPs as Biomarkers in Endometriosis

One study has suggested that MMP3 may be one of the most significant genes of the 291 genes that are responsible for endometriosis [105]. Collectively, MMPs are important in the progress of endometriosis. A cross-sectional study found higher MMP2 expression in the sera of women with stage III/IV endometriosis than in women with stage I/II endometriosis, although there were no differences between women with and without endometriosis [106], which may be due to the low sample size (*n* = 30). Another study showed that MMP2 and TIMP2 were associated with advanced-stage endometriosis [107]. In addition, Wu and other experts found that the expression of MMP9 was increased in patients with recurrent ovarian endometriosis [108]. The expression of MMPs could have a certain reference value for judging the stage of the disease. However, there have been few clinical studies in this area; larger sample sizes and more rigorous experimental protocols are required to verify this.

### 5.2. MMPs as Therapeutic Targets

The current medical therapies may be classified into two groups: surgery and conservative medication. Surgery is effective but can be traumatic. Gonadotropin-releasing hormone (GnRH) agonists and synthetic progestins are commonly used in clinics; however, they can cause numerous systemic side effects due to the over-suppression of endogenous steroid hormone levels [109]. Most patients show severe menopausal symptoms, such as hot flashes, night sweats, sexual hypoactivity, and osteoporosis [110]. Thus, there are still challenges in the treatment of endometriosis, and it is urgent to investigate additional optimal therapies.

MMPs are required for endometrial cells to detach from the endometrium and invade the peritoneum surface, for vascular endothelial cells to migrate to the new vessel, for macrophages to recognize and phagocytose escaped cells, and for NK cells to kill targeted cells, suggesting that MMPs may be an important target for treating endometriosis. To date, several types of MMP inhibitors have been applied to treat malignant tumors. As mentioned previously, TIMPs are natural inhibitors of MMPs. Arkadash et al. modified a high-affinity and highly specific inhibitor of MMP14, which is a TIMP analog [111]. Additionally, numerous monoclonal antibodies against MMP9 or MMP14 have been evaluated in clinical trials for gastric and gastroesophageal junction adenocarcinoma, ulcerative colitis, and breast tumors [112,113,114]. However, various side effects have followed. Marimastat (BB-2516) showed much promise in a preclinical setting and reached phases II and III for numerous tumors. However, many patients could not ignore the “musculoskeletal syndrome”, such as stiffness, inflammation, and joint pain, which forced them to eventually discontinue their participation in the study [115]. The anti-metastatic activity of Neovastat (AE-941) depends on its inhibitory effect on MMPs’ enzymatic activity [116]. However, patients could not tolerate its adverse effects, including flatulence, diarrhea, nausea, constipation, and rashes [117]. Although their final results may be unsatisfactory due to the intolerable side effects, their value is evident. The development of inhibitors that are specific for certain MMPs but do not cross-react with other MMPs is critical for the development of future MMP inhibitors.

Surprisingly, few studies have focused on the application of anti-MMP compounds for patients with endometriosis. In endometriosis, the levels of TIMPs are controversial, but this has not prevented studies from demonstrating that promoting their expression can suppress endometrial cell invasion. Most studies regard MMPs as biomarkers and have proven that medicine has the ability to repress the expression of MMPs. However, the specific mechanism by which MMPs function is yet to be elucidated. Sharpe et al. injected 40 female Sprague-Dawley rats with GnRH-a and a diluent. After examining the peritoneal fluid, their results suggested that GnRH-a altered the activity of MMPs [118]. Moreover, it has been reported that dehydrocostus lactone, isolated from *Aucklandia lappa*, inhibited the expression of MMP2 and MMP9 in endometriosis-associated macrophages (EAMs) and influenced the polarization of macrophages [119]. Resveratrol exerts its anti-inflammatory effect by modulating MMP activity [26]. Furthermore, cisplatin combined with letrozole was found to inhibit angiogenesis in a rat model of endometriosis by altering MMP2 activity [120]. As depicted in Figure 1 and Table 2, MMPs have been reported to play a key role in the invasion. However, additional in-depth mechanistic studies should be conducted; in particular, it is necessary to conduct a safety assessment for anti-MMP interventions. Overall, examining numerous medicines has provided evidence to support their anti-endometriosis activity by reducing MMP activity. However, there is no relevant research on MMP antibodies in endometriosis. Studies on this should be conducted in the future.

## 6. Summary and Perspective

At present, endometriosis is a challenge for patients, clinicians, and researchers on account of the poor understanding of how and why the disease develops. As a result, the effects of clinical medication remain unsatisfactory. MMPs have been an intriguing target for decades. However, no MMP antibodies or inhibitors have yet been used in clinical settings due to their intolerable side effects or non-specificity. Hence, further investigation of their mechanisms and inhibitors is required; this will enhance the therapeutic abilities of drugs. In endometriosis, information relevant to anti-MMP treatment is lacking, which poses significant challenges. Considering the crucial role of MMPs in the development of endometriosis, it is easy to see the necessity of exploiting anti-MMP drugs. Endometriosis is a benign disease that behaves in a malignant manner, and it is important to weigh the advantages and disadvantages of its treatment. Therefore, many aspects of using MMPs as a therapeutic target need to be carefully considered: For example, can anti-MMP approaches be used in endometriosis? How can anti-MMP treatments achieve potent effects without impairing gestation? Furthermore, how can inhibitors or antibodies be optimally designed in order to reduce side effects? This review highlights the location, function, and potential value of MMPs, which will help to improve the understanding of the categories and functions of these key enzymes. Moreover, the review aims to promote the optimization of future therapies (i.e., making them more specific and patient-friendly).

## Figures and Tables

**Table 1:** Changes in MMPs in endometriosis.

Classification	MMPs	Location	Change (Endometriosis. vs. Control)	Reference
Collagenases	MMP1	Eutopic endometrium Peripheral blood	up down	[28] [29]
	MMP13	Ectopic endometrium Peritoneal fluid	up down	[30] [31]
Gelatinases	MMP2	Ectopic endometrium Eutopic endometrium Peripheral blood Peritoneal fluid	up up down ns up up	[24,32,33,34,35] [36] [37] [25,34] [24,38] [24,38]
	MMP9	Ectopic endometrium Eutopic endometrium Peripheral blood	up ns down up	[32,33,39] [25] [28] [40]
Stromelysins	MMP3	Ectopic endometrium Eutopic endometrium Peripheral blood	up down ns	[34,35,41,42] [34] [29]
	MMP10	Ectopic endometrium	up	[35]
	MMP11	Ectopic endometrium Eutopic endometrium	up down	[43,44] [34]
Matrilysins	MMP7	Ectopic endometrium Peripheral blood	up up	[43,45,46] [45]
	MMP26	Ectopic endometrium	up	[27]
Membrane-type MMPs	MT1-MMP	Ectopic endometrium Eutopic endometrium Peritoneal fluid	up up down	[33,37] [36] [31]
	MT5-MMP	Eutopic endometrium	up	[47]
Other MMPs	MMP12	Ectopic endometrium	up	[30]
	MMP23	Ectopic endometrium	up	[27]

MMP, matrix metalloproteinase; ns, no significant difference.

**Table 2:** MMP interventions for endometriosis.

Drug(s)	In Vivo or In Vitro	Species or Cell Type	MMP	Function	Reference
GnRH-a	In vivo	Rats	MMPs	Inhibit invasion	[118]
Atorvastatin and amygdalin	In vivo	Rats	MMP2, MMP9	Inhibit invasion	[121]
Jiawei Foshou San	In vivo	Rats	MMP2, MMP9	Inhibit invasion	[122]
Naringeni	In vivo	Rats	MMP2, MMP9	Inhibit invasion	[123]
Nobiletin	In vivo	Mice	MMP1, MMP3	Inhibit invasion	[124]
Euterpe oleracea extract	In vivo	Rats	MMP9	Inhibit invasion	[125]
Doxycycline	In vitro	12Z epithelial endometriotic cells, human endometriotic stromal cells	MMP2, MMP9	Inhibit invasion	[126]
Pueraria flower extract	In vitro and in vivo	Human endometriotic cells and mice	MMP2, MMP9	Inhibit invasion	[127]
Cervus elaphus	In vitro	Human endometriotic cells	MMP2, MMP9	Inhibit invasion	[128]
Cisplatin and letrozole	In vivo	Rats	MMP2	Inhibit angiogenesis	[120]
Dehydrocostus Lactone	In vitro	Human macrophages	MMP2, MMP9	Inhibit activation	[119]
Resveratrol	In vivo	Human (endometrial tissue, fluid, and serum)	MMP2, MMP9	Anti-inflammatory	[26]
Montelukast	In vivo	Rats	MMP2	Decrease the area of lesions	[129]
Curcumin	In vivo	Mice	MMP2,TIMP2, MT1-MMP	Decrease the area of lesions	[130]
1,25-Dihydroxy Vitamin D3	In vitro	Human endometriotic stromal cells	MMP2, MMP9	May inhibit invasion	[131]

MMP, matrix metalloproteinase.

## Data Availability

Not applicable.
